# Supplementary material for: Are carbon emissions trading and green financial instruments synergistic? -Comprehensive quantitative research based on content analysis
Source: PLoS One. 2024 Mar 7;19(3):e0298601. doi: 10.1371/journal.pone.0298601 (PMC10919601; doi:10.1371/journal.pone.0298601)
Supplement: S1 Data — (ZIP) [file pone.0298601.s001.zip › Data/地级市Z_F工作报告.zip!/╣≤╓▌/░▓╦│╩╨/└┤╫╘╣1⁄2╓┌║┼í╛┬φ┐╦╩2╛▌═°í┐_2015.docx]

各位代表：

现在，我代表市人民政府向大会作工作报告，请予审议，并请市政协委员和其他列席同志提出意见。

一、2014年工作回顾

过去一年，在省委、省政府和市委的坚强领导下，在市人大、市政协的监督、支持下，我们团结和依靠全市各族人民，坚持主基调主战略，紧紧围绕提速发展、后发赶超、同步小康的总目标，始终把全面深化改革贯穿于经济社会发展各个领域各个环节，团结奋进、攻坚克难，狠抓工作落实，全力推进工业化、城镇化、农业现代化和旅游产业化“四化”同步发展，实现经济快速增长，民生持续改善，社会事业全面进步，发展迈上新台阶。

——一年来，我们保持定力，抓投资、保增长，经济总量不断壮大

主要指标高位增长，经济发展实现增比进位。预计，全市实现生产总值516.26亿元，增长14.8%，增速从2013年排名全省第六跃居全省第一，实现历史性突破；人均GDP达到22525元；财政总收入84.46亿元，增长20.2%，公共财政预算收入58.48亿元，增长24.7%；税收收入80.67亿元，增长27.3%，增速排名全省第一；金融机构人民币存款余额660.28亿元，增长8.3%，贷款余额518.95亿元，增长20.4%。在全省经济发展综合测评中保持第五位，成功打赢“保位战”。

项目建设快速推进，固定资产投资大幅增长。预计，全社会固定资产投资1000亿元，增长65.6%；50万元以上固定资产投资723.01亿元，增长76.3%，增速保持全省第一。实施463个重大工程和重点项目，完成投资549.05亿元，增长52.5%，其中亿元以上项目269个，完成投资503.48亿元，增长66.2%。先后7次组织项目集中开工，总投资达636亿元。全市项目库投资规模保持在1.35万亿元以上。

城乡收入持续增长，人民生活水平稳步提升。预计，城镇居民人均可支配收入21099元，增长10.3%，农村居民人均可支配收入6660元，增长13.8%，增速均高于全省平均水平；社会消费品零售总额125.79亿元，增长12.8%；居民消费价格指数涨幅2.3%；城镇登记失业率控制在3.14%以内，新增就业人数增长24.4%，增速排名全省第一。

——一年来，我们精准发力，抓平台、强产业，发展后劲不断增强

工业经济提速转型，园区聚集效应凸显。预计，全部工业增加值148.72亿元，增长14.2%，增速排名全省第三；规模以上工业增加值129.29亿元，增长13.5%。产业结构逐步优化，轻工业、民营工业、新兴产业增加值占工业增加值比重分别达21%、64%、5.6%。全年工业投资290亿元，增长61%，32个3000万元以上工业项目建成投产。百灵天台山制药、汇景纸业、晨春石材、华兴玻璃、云马汽车等发展壮大，久联民爆、西南水泥、方大黄果树饮料等相继投产达产，天韵石尚石材、绿色动力垃圾发电、联鑫工艺、金叶薄片等顺利开工，国电南自、软通动力等信息产业项目落户我市。安顺民用航空产业基地、黎阳高新产业园区、西秀产业园区、普定循环工业基地核心区实现“八通一平”。全市建成标准化厂房111.36万平方米，入驻率94.7%，新增入园企业113户，新增就业5000余人。工业发展呈现新态势，镇宁在探索“就地取材”和“无中生有”上取得明显成效，百灵制药围绕科技创新取得新突破。4个开发区在全省开发区综合考评中实现增比进位。

农业发展提质增效，扶贫开发扎实推进。预计，第一产业增加值78.56亿元，增长6.8%，非粮产业增加值占第一产业比重达84.3%。粮食总产量68.3万吨，蔬菜、茶叶、中药材、精品水果种植面积分别达到122.5万亩、44.1万亩、33万亩、50.7万亩，收购烤烟18.1万担，粮经比调整为34︰66。生态畜牧业发展势头强劲，新增省级以上标准化规模畜禽养殖场35个，畜牧业发展速度和质量保持全省前列。省级重点农业示范园区从8个增加到13个，完成投资32亿元，实现总产值35亿元。市级以上农业产业化龙头企业达到145家，农民专业合作社1275个，农机化综合水平45%。紫云获批国家级农业综合开发县。扶贫资金投入进一步加大，争取到财政专项扶贫资金3.42亿元，精准扶贫全面推进，建档立卡工作顺利完成，普定县和东屯、十字、六马、普利、火花等10个贫困乡镇顺利实现“减贫摘帽”。

旅游产业转型升级，景区景点亮点纷呈。预计，全年接待游客3175.16万人次，增长22.9%，旅游总收入307.6亿元，增长23.5%。黄果树旅游升级版成效显著，半边街生态恢复工程全面完成，推出了半边街休闲步道、国际房车营地、商业步行街和石头寨新景，黄果树智慧旅游平台在全省处于领先地位。龙宫龙潭、漩塘景区和格凸河户外休闲设施升级改造顺利完成，云山屯、本寨、天龙大屯堡旅游景区启动建设。大型实景剧《阿依朵》顺利推出。成功举办黄果树瀑布节、油菜花旅游节、坝陵河大桥跳伞国际邀请赛、格凸河攀岩挑战赛、国际半程马拉松赛及“全国名家谈安顺旅游发展升级版”等活动。旧州古镇、云峰屯堡和兴伟石博园荣获国家4A级旅游景区称号。成功召开首届安顺旅游产业发展大会、服务业发展大会，以旅游为龙头的现代服务业加快发展，第三产业实现增加值249.29亿元，增长16.1%，占全市生产总值比重48.3%。

——一年来，我们持续用力，抓统筹、强基础，城乡面貌不断改善

基础设施不断完善，发展条件逐步优化。完成交通运输投资120亿元，增长37.6%。沪昆高铁及境内3个站点建设进度加快，安六铁路开工建设。安木公路、关岭灞陵大道建成通车，织普高速加快建设，安紫高速顺利开工，花安高速前期工作有序推进，建成通村公路1549公里。黄果树机场新开通至昆明、上海航线，全年进出港旅客突破11万人次。完成水利投资26.1亿元，增长51.4%。治理病险水库14座，新增灌溉面积6.23万亩。黔中水利枢纽、石朱桥、新场河水库工程顺利推进，三岔河、老营、猫洞河水库开工建设，黄家湾水利枢纽工程获国家批准立项。安电三期工程前期工作全面完成，获得核准批复。马马崖一级水电站建成投用,永宁、普利、普屯坝风电场加快建设。完成电网建设投资7.75亿元。27个乡镇邮政网点建成运营，实现全覆盖。“三网融合”试点工作稳步推进，4G网络顺利推广，新增145个自然村通电话、210个行政村通宽带。贵阳、遵义、安顺三地电话网并网升位。

城市建设进度加快，服务功能日益完善。完成城镇建设投资386亿元，增长59.2%。贵安大道、安普大道建成通车，平坝、普定与中心城区同城化发展步伐加快。平坝“撤县设区”获国务院批准。二环路、北四号路等骨干路网基本建成，中心城区面积拓展到90平方公里。虹山湖、高铁站片区基础设施建设全面启动，中华西路等棚户区改造扎实推进。市体育中心主体完工，安顺学院改扩建工程、安顺职院新校区建设等项目顺利推进，虹山湖景观整治二期工程启动实施，中心城区重要节点立交改造加快推进。全国文明城市、国家园林城市、国家卫生城市、国家环保模范城市和双拥模范城等创建工作有序推进。

城乡发展统筹推进，人居环境有效改善。2014年，城镇化率达到41.3%。我市成功获批国家新型城镇化综合试点，迎来城乡一体化发展新的重大机遇。万绿城、多彩万象等城市综合体建设运营加快，完成投资30亿元。旧州、夏云、白岩等一批特色小城镇建设成效明显，省、市级示范小城镇“8+X”项目建成103个，完成投资39亿元。“四在农家·美丽乡村”基础设施建设六项行动计划加快推进，创造了镇村联动“旧州经验”。城乡绿化、美化、净化、亮化工程持续推进，城市扬尘、户外广告、油烟污染治理成效明显。大西桥鲍屯、大山大寨、旧州浪塘、黄果树石头寨等一批示范村面貌焕然一新，黄果树滑石哨、龙宫桃子村荣获“中国最美休闲乡村”称号。

生态建设成效明显，环境质量持续向好。完成营造林36万亩，治理石漠化面积89.5平方公里、水土流失面积 150平方公里，森林覆盖率达到44%。中心城区建成区绿地率30.4%，绿化覆盖率34.4%，人均公园绿地面积6.8平方米。划定虹山湖公园和金钟山片区保护区。邢江河国家湿地公园开展试点建设。虹山湖、娄家湖水环境治理稳步推进，贯城河河道整治初见成效，夜郎湖水源地环境保护进一步加强，全市集中式饮用水源地水质达标率99.55%。中心城区污水处理厂三期、垃圾无害化处理工程加快推进，农业面源污染治理力度进一步加大，城镇污水处理率79.4%，城乡生活垃圾无害化处理率63.7%。旧州浪塘生活污水生态处理法凸显良好生态环保效益。完成国家和省下达的节能减排任务，二氧化硫、氮氧化物排放量分别下降14.84%、16.07%，城市环境空气质量达标率93.1%。

——一年来，我们深挖潜力，抓改革、促开放，发展活力不断提升

深化改革蹄疾步稳，先行先试步伐加快。民营经济、金融投资、文化旅游、社会事业等方面改革稳步推进。组建安顺油脂集团等混合所有制企业，新增规模以上民营企业50家，民营经济增加值占生产总值比重达63%。农村土地流转经营权抵押贷款工作顺利启动，普定县发放全省第一本《农村土地流转经营权证》。融资渠道不断拓展，家喻新型材料成功在“新三板”挂牌交易，安顺富海创投和先进装备创投两支基金获批，融资总额5.03亿元，我市成为全国唯一同获两支基金的地级市。农村金融改革稳步推进，成功组建紫云农村商业银行，农信银自助终端实现行政村全覆盖。平坝、普定、关岭、紫云成功创建金融信用县，占全省10个金融信用县的40%。西秀区义务教育学区管理改革试点工作顺利推进。西秀区和黄果树景区、格凸河景区纳入全省旅游体制机制改革试点。龙宫风景区实现所有权、管理权、经营权分离。新增普定、镇宁成为县级公立医院改革试点县。

对外开放步伐加快，招商引资成效显著。与沿海城市、发达地区和对口帮扶城市的交流合作不断加强，与贵阳、贵安新区签订战略合作协议。青岛新一轮对口帮扶工作全面推进，青岛—安顺共建产业园区启动建设。争取各民主党派中央和全国工商联支持，签订多个合作协议。安顺经济技术开发区公用型保税仓库项目成功获批。在广州、成都等地新设立8个招商分局，积极参与酒博会、厦洽会、西博会等系列招商活动，先后赴新加坡、迪拜、台湾、香港等地举行招商引资推介，成功引进了中核工业、广药集团、国电集团等一批500强企业来安投资。招商引资实际到位资金1016亿元，增长31.9%，其中省外境内到位资金403.1亿元，增长17.9%。实际利用外资1.02亿美元，增长27.4%，外贸进出口总额1.45亿美元，增长3.7倍。

——一年来，我们凝心聚力，抓保障、惠民生，人民福祉不断增进

公共服务日趋完善，社会事业全面进步。教育“9+3”计划和“4+2”突破工程加快推进，中职学校“百校大战”项目进展顺利，学前三年毛入学率和高中阶段毛入学率超过全省平均水平。综合科技进步水平指数53.3%，每万人人才资源数892人，均排名全省第三。实施国家、省科技项目141项，全市获得专利授权799件，12家单位获得省级知识产权试点示范项目。成功举办第二十四届全国图书交易博览会安顺分会场系列活动，“虹湖音乐季”成为备受关注的公共文化品牌，云山屯纳入全国50个传统村落文化遗产保护计划，普定县被命名为“全国文化先进县”。建成15个乡镇级、308个村级农民体育健身工程，在全省第八届少数民族传统体育运动会和第五届残疾人运动会上取得优异成绩。医疗卫生事业加快发展，基本公共卫生服务均等化水平全面提高。人口计生工作扎实推进，全面完成“双降”目标任务。国防教育、民族、宗教、档案、史志、外事、侨务、对台、人防、慈善、气象、水文、防震减灾、质量技术监督、粮食安全保障、住房公积金管理、关心下一代等工作取得新成绩。

综合治理切实加强，和谐社会稳步推进。扎实推进“平安安顺”建设，深化社会治安综合治理，严厉打击违法犯罪，社会和谐稳定局面进一步巩固，全市群众安全感指数达96.87%，提高3.24个百分点，关岭自治县群众安全感在全省排名第一，西秀区在全省中心城区排名第一，并被民政部命名为“全国和谐社区建设示范城区”，塔西社区、虹轴北社区被命名为“全国和谐社区建设示范社区”，华西派出所荣获“全国公安机关爱民模范集体”荣誉称号。社区矫正工作进一步加强，西秀、镇宁、关岭特殊人群康复基地建设不断加快，工读学校启动建设。全面完成第九届村（居）委会换届选举工作。扎实开展“重大信访矛盾和信访问题集中化解百日攻坚战”，特殊疑难信访件化解率达98.9%。安全生产责任体系不断完善，实现安全事故起数和死亡人数“双降”目标。

十件实事基本完成，民生事业持续改善。2014年十件惠民实事共涉及25项具体工作。目前，新建保障性住房2.8万套、新增4000个公共停车位、搬迁扶贫生态移民1.1万人、减少农村扶贫对象10万人、实施10万人安全饮水工程、提高城镇居民医保和新农合补助标准、实施广播电视“村村通”工程2.5万户、提高城乡最低生活保障标准、新建8个以上留守儿童之家、新增城镇就业4.6万人、实施中心城区山体公园化绿地建设工程、人口计生利益导向“四项制度”覆盖目标人群10万人（户）、开通双层观光公交和中心城区至普定公交等23项具体工作已全面完成。2项具体工作尚未全面完成，一项是10条断头路还差2条未打通，中华西路二期和横一路一期道路工程正在推进中；另一项是中心城区至平坝公交尚未开通，待贵安大道辅道和公交站台等设施完善后开通。

过去的一年，我们切实加强政府自身建设，扎实开展党的群众路线教育实践活动，加大反腐倡廉力度，加强行政监察和审计监督，努力做到勤政、廉洁、务实、高效。取消行政许可和非行政许可事项15项，下放79项。电子政务建设加快推进。应急管理工作进一步加强，预防处置突发公共事件的能力得到提高。自觉接受人大及其常委会的法律监督和工作监督，自觉接受政协的民主监督，办理人大代表建议69件、政协提案227件，满意率100%。认真听取各民主党派、工商联和无党派人士的意见建议，积极支持工会、共青团、妇联、文联、科协、残联、红十字会等人民团体依法按章履行职责。

各位代表，过去的一年，通过全市上下的共同努力，我们各项工作都取得了显著成绩，全市地区生产总值增速等4项指标全省排名第一，经济发展群众满意度全省排名第二；我们成功承办全省第三届小城镇建设发展大会、第三次项目建设现场观摩会等全省性大会；我市成功评为省级文明城市、省级园林城市，获得全国文明城市提名；省委、省政府主要领导多次对安顺的工作给予了充分肯定。这些成绩的背后，反映了全市广大干部群众思想观念的重大转变、工作作风的重大转变、精神状态的重大转变。2014年，全市最大的变化就是精神面貌为之一振，大家的注意力、兴奋点都聚焦到了发展上；最大的收获就是统一了思想、凝聚了共识，增强了提速发展、后发赶超、同步小康的坚定信心和决心。这一刻，我们比任何时候都深深地认识到：有信心就有勇气，有信心就有力量，信心比黄金更重要！

回顾过去一年的工作，我们深切体会到，在国内、省内经济下行压力加大的情况下，我市能够实现持续快速发展、增比进位，成绩来之不易，这是省委、省政府和市委坚强领导，市人大依法监督、市政协民主监督与支持的结果，是全市广大干部群众及社会各界同心同德、艰苦奋斗的结果。在此，我谨代表市人民政府，向全市各族人民，向各位代表、政协委员，向各民主党派、工商联、无党派人士、人民团体，向驻安人民解放军、武警消防官兵、公安政法干警、中央和省驻安单位，向老同志及所有关心、支持我市改革发展和现代化建设的各界人士，表示衷心的感谢并致以崇高的敬意！

在看到成绩的同时，我们也清醒地认识到存在的问题和不足，主要表现在：一是经济总量小、人均水平低等问题仍没有得到根本性的解决和改变，部分主要经济指标与其它兄弟市州相比仍有不小差距；二是结构调整任重道远，主导产业带动力不强，缺乏大项目支撑，资源环境压力增大，节能减排和环境保护形势紧迫；三是民生投入不足，涉及群众切身利益的许多问题尚未得到很好解决，扶贫攻坚任务艰巨，社会治理水平有待进一步提高；四是政府应对新形势新变化新常态的能力亟待加强，工作效率不高，发展环境还需进一步优化；五是少数干部缺乏担当意识，执行力和落实力不强，存在“推、拖、躲”的现象，作风建设还需进一步加强，等等。为此，我们将正视存在的问题，进一步提振精气神，增强自信心，咬定目标不放松，凝心聚力抓落实，通过更好更快发展来解决我们前进中遇到的困难和问题。

二、2015年重点工作

2015年，是全面完成“十二五”规划的收官之年，是全面深化改革的关键之年，也是全面推进依法治国的开局之年，做好今年的各项工作意义重大。我们将在省委、省政府和市委的坚强领导下，紧紧围绕提速发展、后发赶超、同步小康的总目标，坚持“稳中求进、进中求快、快中保好、又好又快”的总要求，坚守发展和生态两条底线，主动适应新常态，抓住用好新机遇，认真谋划“十三五”，以开展国家新型城镇化综合试点和承办第十届贵州旅游产业发展大会为抓手，全力以赴打好“一二四”攻坚战，深化改革，扩大开放，推进依法治市，保障改善民生，努力实现全市经济社会整体发力、快速发展。

2015年，全市经济社会发展的主要目标是：地区生产总值增长14%，力争达到16%；规模以上工业增加值增长15%；全社会固定资产投资增长45%以上；公共财政预算收入增长20%以上；社会消费品零售总额增长14%左右；招商引资实际到位资金增长45%；城镇居民人均可支配收入增长13%，农村居民人均可支配收入增长16%；城镇登记失业率控制在4.2%以内；人口自然增长率控制在5.7‰以内；居民消费价格指数涨幅控制在3%左右；完成国家和省下达的节能减排目标任务。

围绕实现上述目标，重点抓好以下八个方面工作。

（一）整体发力狠抓固定资产投资，全面提升经济总量

全力加快项目建设。继续实施项目带动战略，确保50万元和500万元以上固定资产投资分别增长40%以上，增速保持全省第一。进一步调整优化投资结构，产业投资占固定资产投资比重达到35%以上。市级筹措项目前期经费5000万元以上，扎实推进项目前期工作，充实完善项目库，确保项目库投资规模保持在1.38万亿元以上。切实抓好重点行业、重点领域项目建设，实施市级重大工程和重点项目452个，年度投资733亿元以上。进一步加强项目调度和督促检查，实行市级领导包保责任制，确保项目建设顺利推进。完善项目入库要件手续，确保应统尽统。

全力抓好要素保障。最大限度争取金融机构贷款资金支持，确保银行业金融机构存贷款余额增幅高于全省平均水平。拓宽项目融资渠道，完善市场化投融资机制，鼓励支持企业直接融资，积极开展发行地方债工作。推进融资企业转型升级，打造一批集资本运作、实体经营为一体的国有企业。加快推动政府与社会资本合作，积极吸收社会资金参与项目建设。科学处理保护耕地与保障用地关系，积极推进集约节约利用土地，清理盘活存量土地，新增建设用地500公顷。

全力夯实发展基础。深入实施综合交通建设三年攻坚行动，完成交通运输建设投资130亿元。加快建设安紫、织普、花安高速公路和马厂至天星桥公路，开工建设木山堡至紫云、顶云至盘江桥、丁旗至木岗等公路，力争宁谷至黄果树一级公路、幺铺至镇宁城市快速干道、黄桶至幺铺大道、关岭至花江大道开工，建设1130公里通村油路（水泥路）。开工建设中心城区北客运站和安顺西综合客运枢纽站。扎实推进光照、马马崖、董菁、夜郎湖、引子渡库区航运基础设施建设。完成铁路建设投资20亿元，基本完成沪昆高铁安顺段及境内3个站点建设，加快推进安六铁路建设。积极推进黄果树机场第二跑道建设相关工作，加大航线培育力度，力争开通2条以上新航线，实现进出港旅客20万人次。深入实施水利建设三大会战，完成50万元以上水利建设投资30亿元。启动木拱河、红坪、雨棚等小型水库建设，力争黄家湾水利枢纽工程、大洞口水库、偏坡水库动工建设。治理病险水库和重点山塘18座，继续做好地下水开发利用工程，建设机井200口。完成电网建设投资4.5亿元。推进风力发电。实施信息基础设施建设三年会战，完成信息基础设施建设投资8.8亿元。加快电信网、互联网、广播电视网“三网融合”。

（二）整体发力狠抓招商引资，全面提升改革开放水平

突出招商力度。创新招商引资方式，大力开展小分队招商、驻点招商、平台招商、以商招商，鼓励全民招商，形成“大招商”格局。坚持招大商、选好商，瞄准世界500强、中国500强及上市公司，围绕装备制造、民用航空、新医药大健康、特色轻工、石材加工、新型材料、电子科技、大数据等产业开展招商引资工作，切实增强招商引资工作的针对性和实效性。积极参加第十一届“泛珠会”、第五届中国（贵州）国际酒类博览会、贵州·香港投资贸易活动周等大型对外交流活动。进一步优化招商引资环境，加大项目跟踪服务力度，大力营造亲商、安商、便商、护商的良好氛围。

突出开放广度。围绕加快建设黔中经济区，深化与贵阳市、贵安新区的战略合作，推进贵安一体化发展。全面融入长江经济带、泛珠三角经济区，进一步加强与沿海发达地区的交流合作。紧抓青岛全方位对口帮扶机遇，高起点、高标准建设青岛—安顺产业园区。充分利用高铁时代带来的人流、物流、资金流、信息流新机遇，大力发展高铁经济，拓展对外合作空间。抢抓“一带一路”战略机遇，着力构建内陆开放型经济新高地，积极加强与东盟、中东、韩国和台湾地区的交流，加快推进“安新生态产业园”建设。深入实施外贸进出口翻番计划，完成进出口总额1.74亿美元。

突出改革深度。深入推进重点领域和关键环节体制机制改革，破解制约发展的瓶颈。支持国有企业转型升级，加快推进混合所有制经济发展，全面落实民营经济与国有经济同等待遇的各项政策措施，鼓励国有、民营经济互相参股，促进多种经济成分共生繁荣。深入实施提高民营经济比重五年行动计划，新增规模以上民营企业55家，扶持微型企业1600户，民营经济增加值占生产总值比重64%以上。加快农村金融改革，组建关岭农村商业银行，争创农村金融信用市。积极有序推进农村土地承包经营权确权登记颁证工作，促进农村土地合理流转，推动经营权抵押贷款。开展集体经营性建设用地流转试点，探索农村宅基地有序退出机制，加快建立城乡统一的建设用地市场。深化农村小型水利工程产权制度改革，开展农业水价综合改革试点工作。推进西秀区和黄果树景区、格凸河景区旅游体制机制改革试点工作，深化重点旅游景区经营管理体制改革，采取特许经营权转让、合资合作、股份制等方式，引进战略投资者参与旅游景区建设。着力推进民生领域的改革发展，支持学校、医院、文艺团体等引进民间资本合作发展。

（三）整体发力推进新型工业化，全面增强实体经济实力

加快工业转型升级。大力发展航空、特色轻工、能源、原材料、战略性新兴产业“五大支柱产业”，构建多元支撑的现代产业体系，力争50万元以上工业投资390亿元，增长34%以上，实现全部工业增加值180亿元，增长14%，规模以上工业增加值160亿元，增长15%。围绕把石材产业打造成全省“第六张名片”，着力推进石材产业发展，建设西南最大的石材交易博览中心，积极申报建设省级石材产品检验检测中心。推动军民深度融合，发展壮大装备制造业。充分发挥独特的中药材资源优势和良好的气候生态优势，加快发展新医药大健康产业。围绕大数据产业，以“7+N”云工程和“云上贵州”系统平台建设为契机，努力促进信息产业发展，支持软通动力云服务平台、国电南自数据中心、青岛禾软科技等项目实施。突出抓好重点行业、重点企业的节能管理和技术改造，逐步淘汰落后产能，确保万元地区生产总值能耗降低3.09%，工业固体废物综合利用率提升到85%以上。

实施园区成长工程。理顺园区管理体制，实施园区梯队培育计划，努力培育百亿级、千亿级园区。加快推进安顺经济技术开发区申报国家级开发区，启动黎阳高新技术产业园区申报国家级高新技术产业园区工作。推进园区基础设施建设，加快环保设施建设，提高标准化厂房入驻率，提升园区项目承载能力和持续发展能力。做大做强安顺民用航空产业基地、西秀产业园、黎阳高新技术产业园、普定循环工业基地等园区，加快建设镇宁石材产业园、新医药大健康产业园等特色园区。全市园区完成基础设施投资115亿元，产业项目投资165亿元，工业总产值550亿元。

扶持企业做大做强。加强工业经济运行监测，确保煤、电、油、运等要素保障，促进企业满负荷生产经营。以“双服务”和“百千万”工程为抓手，帮助企业解决生产经营中的困难和问题，促进现有企业释放产能和在建项目投产达产。支持贵飞公司打造百亿级企业，建设国内最大的无人机生产服务基地，支持云马汽车项目做大做强，支持百灵集团打造50亿级企业。加快恒远建材、联鑫工艺、晨春石材等项目建设进度，推动安电三期尽快开工，建成3000万元以上工业项目50个。培育壮大煤矿企业集团，大力推进煤矿采掘机械化，确保煤炭产量1000万吨、发电量85亿千瓦时以上。大力实施品牌战略，支持企业加大科技创新投入和技术升级改造，优化产品结构。帮助企业开拓市场，实现工业产品产销率95%以上。

（四）整体发力推进新型城镇化综合试点，全面激发城镇发展活力

大力推进新型城镇化综合试点。以破解投融资机制难题为突破口，有序启动城乡一体化、行政区划、多规融合等领域改革，构建有利于新型城镇化健康发展的政策和制度体系。探索建立农业转移人口市民化成本分担机制，全面放开落户限制，有序推进租房或购房、就业或创业农业转移人口落户城镇。探索建立多元化、可持续的城镇化投融资机制，放宽城镇市政公用事业市场准入，加快向民间资本推出投资项目，开展城市基础设施建设PPP试点。建立完善生态补偿和修复机制，推进镇宁、关岭、紫云、普定开展贵州省南部生态补偿示范区建设。加快平坝区设立相关工作，推进普定“撤县设区”，优化市辖区规模和结构，努力建设山水田园型、绿色生态型、宜居宜游型、开放创新型和历史文化型“五型城市”。2015年，全市城镇化率达到44%。

大力推动产城景融合发展。统筹城镇化与工业化、信息化、农业现代化和旅游产业化同步发展。加快城市总体规划修编，推进“四规合一”，坚持产业发展、城镇建设、土地利用和生态环保“一张图”规划。探索建立镇园合一、产城融合的管理模式，通过园区发展实现人口聚集，通过城镇规划建设为园区提供金融、教育、医疗、文化等公共服务，以产兴城、以城促产。加快虹山湖、历史文化街区等城市景点开发，推进以虹山湖、娄家湖、贯城河为重点的城市景观水环境综合治理，不断完善城市功能，彰显城市特色，提升文化品位和旅游城市形象。加快推进城镇慢道、水道、轨道“三道”规划建设，形成各具特色的慢行系统、水系统和轨道系统，把城市、城镇、景区和乡村景点连接起来，实现景城互动。

大力加快城镇建设步伐。加快虹山湖、杨湖、高铁站片区等城市新区建设，进一步拓展中心城区规模。强力推进中华西路二期等城市棚户区和城中村改造项目，加快推进市规划展览馆、市文化馆、市工人文化宫建设，建成市体育中心、市博物馆。完成市污水处理厂三期工程，启动中心城区雨污分流管网建设。打造平坝高铁新区、普定“朗泊湾”、镇宁环翠新区、关岭顶云新区、紫云新城等一批县城重点新区。加快示范小城镇建设，完成“8个1”和“8+X”项目投资22亿元以上。推广“旧州经验”，加快建设一批特色示范小城镇。大力发展建筑业，扶持本地建筑企业做大做强。

大力提升城市管理水平。多形式加强宣传引导，调动全社会力量共同治理不文明行为，提升市民文明素质。加强城市交通管理，优先发展公共交通，严格出租车营运管理，规范车辆停放，缓解重要节点拥堵现象。加大占道经营、噪音污染等治理力度，推广使用清洁能源，降低生活用煤污染。加强公路沿线、城乡结合部、城区背街小巷综合治理，有效整治“脏乱差”现象。依法加大违法用地、违法建设打击力度，形成常态化机制。加大创建工作力度，力争成功创建国家环保模范城市和全国双拥模范城，积极申报国家历史文化名城。

（五）整体发力做优山地现代高效农业，全面促进农村可持续发展

加快农业产业结构调整。转变农业发展方式，不断提高土地产出率、资源利用率、劳动生产率，实现集约发展、可持续发展。种植粮食180万亩，确保粮食产量65万吨，种植蔬菜135万亩，新增种植水果8.8万亩、中药材7万亩、茶叶6.4万亩，收购烤烟23万担，粮经比调整到30︰70。加快推进山地生态畜牧业发展，实现肉蛋产量19万吨、水产品产量1.5万吨以上。加快农业园区建设，实现乡镇园区全覆盖。重点推进13个省级现代高效农业示范园区建设，在沪昆、惠兴、安普高速公路及国道、省道沿线加快建设生态观光和特色农业产业带。推进全国丘陵山地农业机械化示范区建设，农机化综合水平达到50%。加快农业科技成果推广应用，加大基层农业技术服务力度。认真组织开展“三品一标”认证工作，积极发展油菜籽、蔬菜、茶叶、金刺梨等农产品精深加工，提高附加值。

努力促进农民增收。认真落实好国家各项强农惠农富农政策，规范涉农项目实施，强化农民负担监管。大力发展农民专业合作社、家庭农场等新型农业经营主体，提高农民组织化程度，扶持龙头企业140家。加大农业服务体系建设，促进农产品大流通，探索建立优势特色农产品电子商务服务平台。鼓励农民创业和务工，大力实施“阳光工程”、“雨露计划”等培训项目。积极发展庭院经济、休闲农业、乡村旅游，多渠道增加农民收入。

加强生态环境建设。深入实施造林绿化三年攻坚战，继续推进退耕还林、天然林保护、珠江防护林等林业重点工程建设，完成营造林36万亩，治理石漠化面积102平方公里，治理水土流失面积160平方公里，森林覆盖率提高到46%以上。加强水资源管理，落实最严格的水资源管理制度，加大集中式饮用水源地保护力度。强化农业面源污染治理，加快推进农村改水、改灶、改厕、改圈，完善城乡垃圾收运系统和污水处理设施。扎实开展“绿化美化乡村”活动，加快沪昆高速沿线造林绿化和村庄景观整治。推进“四在农家·美丽乡村”基础设施建设六项行动计划，打造具有地域特色、充满活力的新农村。

（六）整体发力打造旅游产业升级版，全面促进现代服务业发展

加快旅游产业发展。全力承办好第十届贵州旅游产业发展大会，召开第二届安顺旅游产业发展大会，打造安顺旅游产业升级版。加快第十届旅发大会主会场、观光小火车、旧州“山里江南”、黄果树西游文化休闲步道、邢江河湿地公园环线、普定百花欢乐大世界等一批重点项目建设。加快高星级酒店、智能养老基地、精品客栈、自驾车营地建设。迎接“高铁时代”的到来，加快构建“快进慢游”服务体系，完善旅游交通标识标牌，建设游客集散中心、服务中心，力争开通黄果树、龙宫等景区至贵阳、安顺中心城区的旅游专线，提高旅游通达性。加强行业管理，规范旅游市场秩序，推进旅游标准化建设。强化宣传推介和市场营销，大力开拓国内外旅游市场，全年接待游客4286万人次以上，增长35%，旅游总收入416亿元以上，增长35%。

拓展旅游产业外延。加快推进旅游、文化、体育等业态融合发展，打造特色文化体育旅游精品。围绕屯堡文化，打造一台大型实景舞台剧。启动坝陵河户外运动基地建设。积极争取航空飞行营地项目落户安顺。举办黄果树瀑布节、国际格凸攀岩节、国际马拉松、坝陵河大桥跳伞等重要活动。加快推进黄果树、格凸河、屯堡、关岭地质公园打捆申报世界文化和自然双遗产地。引导建设旅游商品、特色美食、购物娱乐街区。加快旅游信息网络平台和乡村智慧旅游建设。依托农业产业园区和地方民族风情资源，打造一批乡村旅游产品。

推动现代服务业发展。大力实施服务业发展“2125”行动计划，加快发展金融、现代物流、信息技术、商务服务等生产性服务业，大力发展生态文化旅游、健康服务、家庭服务等生活性服务业。加快黄桶-幺铺物流园区建设。积极推进西秀区国家信息消费试点工作。合理布局商业网点，建立健康有序的商品市场体系。深入实施“万村千乡市场”、“农超对接”等工程，推动商贸服务向乡村延伸。全年服务业增加值增长16%以上。

（七）整体发力保障改善民生，全面提升群众幸福指数

加快推进精准扶贫。积极探索扶贫开发新办法新途径，做到结对帮扶、产业扶持、教育培训、农村危房改造、扶贫生态移民、基础设施“六个到村到户”。加大扶贫资金投入，深入实施滇桂黔石漠化片区区域发展与扶贫攻坚工程。大力推广精准扶贫普定“猴场模式”，积极支持镇宁申报“扶贫攻坚示范县”，推进关岭和10个贫困乡镇实现“减贫摘帽”。西秀、平坝在全市率先建成全面小康社会。

全面推进社会事业。加快基本普及15年教育和县域内义务教育基本均衡，继续实施教育“9+3”计划，启动实施第二期学前教育三年行动计划和高中阶段突破工程。推进教育资源合理配置，大力提升教育教学质量。支持安顺学院通过教育部本科教学合格评估，确保安顺职院新校区建成投用。加强人才队伍建设，加大知识产权保护力度，提升科技成果转化能力。弘扬国学文化，举办孔子文化节。加强非物质文化、民俗文化传承、保护与开发，推动文化惠民工程，加快发展文化产业，创新本土文化产品，繁荣文化消费市场。大力开展全民健身运动，筹备贵州省第三届农民运动会，建成市级市民健身中心和2个县级体育场（馆）。推进公立医院改革，发展混合所有制医院，鼓励社会办医。促进医疗资源向县乡基层延伸，实施社区卫生服务机构、乡镇卫生院、村卫生计生室提升工程。确保市人民医院新院、市儿童医院建成运营，推动市中医院创建“三甲”医院。加强人口计生工作，强化新生儿出生缺陷干预和农村妇女健康检查，确保人口出生率和自然增长率实现“双降”。大力推进创业型城市创建工作，以创业带动就业。实施全民参保登记计划，落实好养老保险、医疗保险、新农合和失地农民社会保障政策，推进社会保险制度全覆盖。继续抓好“保供稳价”、“菜篮子工程”，确保居民消费价格水平保持总体稳定。做好全国1%人口抽样调查、第二次全国地名普查工作。

加大社会治理力度。深入开展“平安安顺”创建活动，完成“天网”三期工程建设，加大禁毒工作力度，加强社区矫正工作，依法严厉打击各类违法犯罪活动,进一步提高人民群众安全感和满意度。切实加强城乡社区服务体系建设，努力提高城乡社区综合服务设施覆盖率。加强流动人口和特殊人群服务管理。认真做好新形势下群众工作，完善重大决策、重大项目建设社会稳定风险评估机制，加强矛盾纠纷突出问题和信访案件化解力度。创新社会治理，积极推广“上关经验”。加强公共法律服务体系建设，进一步扩大法律援助覆盖面。牢固树立安全生产“红线”意识，强化企业主体责任，扎实抓好交通运输、建筑、消防、煤矿等重点领域专项整治，确保安全事故起数和死亡人数“双降”。强化食品药品安全监管。加强应急处置能力建设。

切实办好“十件实事”。（1）开工建设保障性住房4.5万套，其中棚户区改造3.3万户；改造农村危房1万户。（2）减少农村扶贫对象10万人；完成扶贫生态移民搬迁1.28万人；解决农村5万人饮水安全问题。（3）城乡居民最低生活保障标准在2014年基础上提高10%以上；建设农村幸福院10个、社区居家养老服务中心20个；免除全市户籍人口基本殡葬服务费。（4）计划生育利益导向“四项制度”惠及14万户（次）；为1.35万对计划怀孕夫妇实施免费孕前优生健康检查。（5）新增城镇就业5万人，新增创业2000户，创业带动就业8000人；新建中心城区综合劳务市场2个。（6）完成20所受地质灾害危害的学校搬迁治理。（7）实现所有乡镇垃圾转运站和示范村垃圾收集站、收运队伍全覆盖；建成15个乡镇和20个示范村污水处理设施。（8）完成中心城区山体公园建设2个，小型游园建设7个，新增公共绿地面积90万平方米。（9）新增700个监控摄像头、90个智能报警点；新打通中心城区10条断头路，完善所有道路路名及交通标识标牌。（10）新建中心城区立体式停车位1200个；新增公交线路2条。

（八）整体发力建设法治政府，全面提升依法治市能力

坚持依法行政。全面履行政府职能，严格按照法定权限和法定程序行使权力、履行职责，加快建设职能科学、权责法定、执法严明、公开公正、廉洁高效、守法诚信的法治政府。坚持法定职责必须为、法无授权不可为，切实保障公民、法人和其他组织合法权益。推行依法决策，把公众参与、专家论证、风险评估、合法性审查、集体讨论决定作为重大行政决策法定程序。积极推行政府法律顾问制度，建立重大决策终身责任追究制度及责任倒查机制。改革行政执法体制机制，推行综合执法。严格规范公正文明执法，全面落实行政执法责任制，严格执法主体责任和执行责任追究制。深入开展“六五普法”，增强全民法治观念，提高公民法律素质，营造良好法治环境。

强化监督制约。深化政务公开，坚持公开为常态、不公开为例外，依法及时、准确地向社会公开政府信息，重点推进财政预算、公共资源配置、重大建设项目批准和实施、社会公益事业建设等领域的政府信息公开。推行政府权力清单制度，建立完善依法审核、发布和动态管理工作机制，依法公开权力运行流程。自觉接受市人大及其常委会的法律监督和工作监督，主动接受市政协的民主监督，认真办理人大代表建议和政协提案。充分听取各民主党派、工商联、无党派人士和人民团体的意见建议。强化监察、审计等内部监督，自觉接受社会监督和舆论监督。

切实转变作风。进一步巩固和拓展党的群众路线教育实践活动成果，坚持不懈纠正“四风”，忠实践行“三严三实”，扎实开展“2651”工程。主动适应新形势、新变化、新常态，切实增强机遇意识、担当意识、创新意识、竞争意识，不断提高政府执行力和落实力。深化行政审批制度改革，精简审批事项，压缩审批时限，提高办事效率。加强效能监察，加大问责力度，严肃查办“庸懒散慢浮”和“冷硬横推拖”等行为，坚决纠正“不作为、慢作为、乱作为”的现象，切实解决以权谋私、与民争利等损害国家和群众利益的问题。

加强廉洁自律。认真落实中央八项规定和省委十项规定，严格执行党风廉政建设责任制，全面推进惩治和预防腐败体系建设。严格控制“三公”经费支出，认真执行厉行节约反对浪费条例。加强工程建设、公共资源交易等重点领域和关键环节廉政监管，从源头上防治腐败。大力营造风清气正的政务环境，以勤政廉洁的形象取信于民。

各位代表，艰辛成就伟业，奋斗铸就辉煌！安顺正处于提速发展、后发赶超、同步小康的全力冲刺时期，我们时刻感受到时间的紧迫、群众的期盼、责任的重大、使命的光荣，让我们更加紧密地团结在以习近平同志为核心的党中央周围，在市委的坚强领导下，实干苦干、砥砺奋进，为实现安顺新的历史性跨越而努力奋斗！
